# Supplementary material for: Somatic Hypomethylation of Pericentromeric SST1 Repeats and Tetraploidization in Human Colorectal Cancer Cells
Source: Cancers (Basel). 2021 Oct 26;13(21):5353. doi: 10.3390/cancers13215353 (PMC8582499; doi:10.3390/cancers13215353)
Supplement: Supplementary file 1 [file cancers-13-05353-s001.zip › cancers-1346744-supplementary-author confirmation-/GSEA.zip!/GSEA_TETRvsDIP/heat_map_corr_plot.html]

Heat map and correlation plot for eset\_byprobe\_collapsed\_to\_symbols.Diploid\_vs\_Tetraploid.cls#Tetraploid\_versus\_Diploid  

Fig 1: heat\_map      
 Heat Map of the top 50 features for each phenotype in eset\_byprobe\_collapsed\_to\_symbols.Diploid\_vs\_Tetraploid.cls#Tetraploid\_versus\_Diploid

  
  

Fig 2: Ranked Gene List Correlation Profile      
 Ranked list correlations for eset\_byprobe\_collapsed\_to\_symbols.Diploid\_vs\_Tetraploid.cls#Tetraploid\_versus\_Diploid

  
  
    
