# Supplementary material for: Somatic Hypomethylation of Pericentromeric SST1 Repeats and Tetraploidization in Human Colorectal Cancer Cells
Source: Cancers (Basel). 2021 Oct 26;13(21):5353. doi: 10.3390/cancers13215353 (PMC8582499; doi:10.3390/cancers13215353)
Supplement: Supplementary file 1 [file cancers-13-05353-s001.zip › cancers-1346744-supplementary-author confirmation-/GSEA.zip!/GSEA_TETRvsDIP/HALLMARK_MYC_TARGETS_V2.html]

Details for gene set HALLMARK\_MYC\_TARGETS\_V2[GSEA]

|  || Dataset | eset\_byprobe\_collapsed\_to\_symbols.Diploid\_vs\_Tetraploid.cls #Tetraploid\_versus\_Diploid.Diploid\_vs\_Tetraploid.cls #Tetraploid\_versus\_Diploid\_repos |
| Phenotype | Diploid\_vs\_Tetraploid.cls#Tetraploid\_versus\_Diploid\_repos |
| Upregulated in class | Tetraploid |
| GeneSet | HALLMARK\_MYC\_TARGETS\_V2 |
| Enrichment Score (ES) | 0.56454176 |
| Normalized Enrichment Score (NES) | 2.0497828 |
| Nominal p-value | 0.0 |
| FDR q-value | 6.75E-4 |
| FWER p-Value | 0.001 |
Table: GSEA Results Summary

  

Fig 1: Enrichment plot: HALLMARK\_MYC\_TARGETS\_V2      
 Profile of the Running ES Score & Positions of GeneSet Members on the Rank Ordered List

  

| SYMBOL | TITLE | RANK IN GENE LIST | RANK METRIC SCORE | RUNNING ES | CORE ENRICHMENT || 1 | UTP20 | UTP20 small subunit (SSU) processome component | 183 | 0.162 | 0.0698 | Yes |
| 2 | SLC29A2 | solute carrier family 29 (equilibrative nucleoside transporter), member 2 | 1017 | 0.084 | 0.0680 | Yes |
| 3 | DCTPP1 | dCTP pyrophosphatase 1 | 1076 | 0.081 | 0.1049 | Yes |
| 4 | NOP16 | NOP16 nucleolar protein | 1218 | 0.078 | 0.1359 | Yes |
| 5 | MCM4 | minichromosome maintenance complex component 4 | 1325 | 0.076 | 0.1674 | Yes |
| 6 | TMEM97 | transmembrane protein 97 | 1371 | 0.075 | 0.2016 | Yes |
| 7 | RRP9 | ribosomal RNA processing 9, small subunit (SSU) processome component, homolog (yeast) | 1374 | 0.075 | 0.2380 | Yes |
| 8 | HSPE1 | heat shock 10kDa protein 1 | 1413 | 0.074 | 0.2721 | Yes |
| 9 | MCM5 | minichromosome maintenance complex component 5 | 1829 | 0.064 | 0.2823 | Yes |
| 10 | LAS1L | LAS1-like, ribosome biogenesis factor | 2029 | 0.061 | 0.3019 | Yes |
| 11 | UNG | uracil DNA glycosylase | 2227 | 0.058 | 0.3201 | Yes |
| 12 | MRTO4 | MRT4 homolog, ribosome maturation factor | 2254 | 0.058 | 0.3469 | Yes |
| 13 | DUSP2 | dual specificity phosphatase 2 | 2355 | 0.056 | 0.3691 | Yes |
| 14 | SORD | sorbitol dehydrogenase | 2500 | 0.054 | 0.3880 | Yes |
| 15 | SLC19A1 | solute carrier family 19 (folate transporter), member 1 | 2746 | 0.051 | 0.4002 | Yes |
| 16 | NPM1 | nucleophosmin (nucleolar phosphoprotein B23, numatrin) | 2890 | 0.049 | 0.4167 | Yes |
| 17 | MYC | v-myc avian myelocytomatosis viral oncogene homolog | 3194 | 0.046 | 0.4234 | Yes |
| 18 | GRWD1 | glutamate-rich WD repeat containing 1 | 3317 | 0.045 | 0.4389 | Yes |
| 19 | PUS1 | pseudouridylate synthase 1 | 3333 | 0.044 | 0.4599 | Yes |
| 20 | WDR43 | WD repeat domain 43 | 3334 | 0.044 | 0.4816 | Yes |
| 21 | WDR74 | WD repeat domain 74 | 3598 | 0.042 | 0.4884 | Yes |
| 22 | NOP56 | NOP56 ribonucleoprotein | 3937 | 0.038 | 0.4897 | Yes |
| 23 | BYSL | bystin-like | 4149 | 0.036 | 0.4966 | Yes |
| 24 | SRM | spermidine synthase | 4706 | 0.031 | 0.4834 | Yes |
| 25 | NDUFAF4 | NADH dehydrogenase (ubiquinone) complex I, assembly factor 4 | 5015 | 0.029 | 0.4819 | Yes |
| 26 | TCOF1 | Treacher Collins-Franceschetti syndrome 1 | 5065 | 0.029 | 0.4935 | Yes |
| 27 | RCL1 | RNA terminal phosphate cyclase-like 1 | 5135 | 0.028 | 0.5038 | Yes |
| 28 | PHB | prohibitin | 5208 | 0.028 | 0.5137 | Yes |
| 29 | MPHOSPH10 | M-phase phosphoprotein 10 (U3 small nucleolar ribonucleoprotein) | 5567 | 0.025 | 0.5075 | Yes |
| 30 | PRMT3 | protein arginine methyltransferase 3 | 5572 | 0.025 | 0.5194 | Yes |
| 31 | PLK4 | polo-like kinase 4 | 5691 | 0.024 | 0.5252 | Yes |
| 32 | NOLC1 | nucleolar and coiled-body phosphoprotein 1 | 5963 | 0.022 | 0.5222 | Yes |
| 33 | PES1 | pescadillo ribosomal biogenesis factor 1 | 6034 | 0.022 | 0.5292 | Yes |
| 34 | IMP4 | IMP4 homolog, U3 small nucleolar ribonucleoprotein | 6086 | 0.022 | 0.5372 | Yes |
| 35 | HSPD1 | heat shock 60kDa protein 1 (chaperonin) | 6186 | 0.021 | 0.5423 | Yes |
| 36 | NOP2 | NOP2 nucleolar protein | 6188 | 0.021 | 0.5524 | Yes |
| 37 | PA2G4 | proliferation-associated 2G4 | 6269 | 0.020 | 0.5583 | Yes |
| 38 | RRP12 | Zhang2013 ALT\_ACCEPTOR, ALT\_DONOR, coding, INTERNAL, intronic best transcript NM\_015179 | 6340 | 0.020 | 0.5645 | Yes |
| 39 | CDK4 | cyclin-dependent kinase 4 | 7198 | 0.015 | 0.5277 | No |
| 40 | CBX3 | chromobox homolog 3 | 7262 | 0.014 | 0.5316 | No |
| 41 | FARSA | phenylalanyl-tRNA synthetase alpha subunit | 7331 | 0.014 | 0.5349 | No |
| 42 | MYBBP1A | MYB binding protein (P160) 1a | 7769 | 0.011 | 0.5180 | No |
| 43 | NIP7 | NIP7, nucleolar pre-rRNA processing protein | 8021 | 0.010 | 0.5099 | No |
| 44 | TFB2M | transcription factor B2, mitochondrial | 8211 | 0.009 | 0.5044 | No |
| 45 | EXOSC5 | exosome component 5 | 8270 | 0.008 | 0.5054 | No |
| 46 | SUPV3L1 | SUV3-like helicase | 8282 | 0.008 | 0.5088 | No |
| 47 | HK2 | hexokinase 2 | 8575 | 0.006 | 0.4970 | No |
| 48 | PLK1 | polo-like kinase 1 | 8734 | 0.006 | 0.4916 | No |
| 49 | DDX18 | DEAD (Asp-Glu-Ala-Asp) box polypeptide 18 | 9630 | 0.001 | 0.4460 | No |
| 50 | GNL3 | guanine nucleotide binding protein-like 3 (nucleolar) | 10216 | -0.002 | 0.4171 | No |
| 51 | IPO4 | importin 4 | 10574 | -0.005 | 0.4010 | No |
| 52 | RABEPK | Rab9 effector protein with kelch motifs | 10945 | -0.007 | 0.3853 | No |
| 53 | MAP3K6 | mitogen-activated protein kinase kinase kinase 6 | 11199 | -0.008 | 0.3763 | No |
| 54 | TBRG4 | transforming growth factor beta regulator 4 | 12791 | -0.018 | 0.3033 | No |
| 55 | NOC4L | nucleolar complex associated 4 homolog | 13212 | -0.021 | 0.2919 | No |
| 56 | PPRC1 | peroxisome proliferator-activated receptor gamma, coactivator-related 1 | 14059 | -0.027 | 0.2615 | No |
| 57 | AIMP2 | aminoacyl tRNA synthetase complex-interacting multifunctional protein 2 | 15543 | -0.039 | 0.2045 | No |
Table: GSEA details [plain text format]

  

Fig 2: HALLMARK\_MYC\_TARGETS\_V2      
 Blue-Pink O' Gram in the Space of the Analyzed GeneSet

  

Fig 3: HALLMARK\_MYC\_TARGETS\_V2: Random ES distribution      
 Gene set null distribution of ES for **HALLMARK\_MYC\_TARGETS\_V2**
