# Supplementary material for: Somatic Hypomethylation of Pericentromeric SST1 Repeats and Tetraploidization in Human Colorectal Cancer Cells
Source: Cancers (Basel). 2021 Oct 26;13(21):5353. doi: 10.3390/cancers13215353 (PMC8582499; doi:10.3390/cancers13215353)
Supplement: Supplementary file 1 [file cancers-13-05353-s001.zip › cancers-1346744-supplementary-author confirmation-/GSEA.zip!/GSEA_TETRvsDIP/HALLMARK_XENOBIOTIC_METABOLISM.html]

Details for gene set HALLMARK\_XENOBIOTIC\_METABOLISM[GSEA]

|  || Dataset | eset\_byprobe\_collapsed\_to\_symbols.Diploid\_vs\_Tetraploid.cls #Tetraploid\_versus\_Diploid.Diploid\_vs\_Tetraploid.cls #Tetraploid\_versus\_Diploid\_repos |
| Phenotype | Diploid\_vs\_Tetraploid.cls#Tetraploid\_versus\_Diploid\_repos |
| Upregulated in class | Diploid |
| GeneSet | HALLMARK\_XENOBIOTIC\_METABOLISM |
| Enrichment Score (ES) | -0.29522502 |
| Normalized Enrichment Score (NES) | -1.2337872 |
| Nominal p-value | 0.09065156 |
| FDR q-value | 0.2610129 |
| FWER p-Value | 0.97 |
Table: GSEA Results Summary

  

Fig 1: Enrichment plot: HALLMARK\_XENOBIOTIC\_METABOLISM      
 Profile of the Running ES Score & Positions of GeneSet Members on the Rank Ordered List

  

| SYMBOL | TITLE | RANK IN GENE LIST | RANK METRIC SCORE | RUNNING ES | CORE ENRICHMENT || 1 | CA2 | carbonic anhydrase II | 69 | 0.250 | 0.0187 | No |
| 2 | FABP1 | fatty acid binding protein 1, liver | 75 | 0.234 | 0.0392 | No |
| 3 | GCNT2 | glucosaminyl (N-acetyl) transferase 2, I-branching enzyme (I blood group) | 76 | 0.233 | 0.0599 | No |
| 4 | CFB | complement factor B | 164 | 0.167 | 0.0703 | No |
| 5 | TTPA | tocopherol (alpha) transfer protein | 185 | 0.161 | 0.0836 | No |
| 6 | FAS | Fas cell surface death receptor | 186 | 0.161 | 0.0979 | No |
| 7 | GSTO1 | glutathione S-transferase omega 1 | 280 | 0.136 | 0.1052 | No |
| 8 | HSD17B2 | hydroxysteroid (17-beta) dehydrogenase 2 | 284 | 0.136 | 0.1171 | No |
| 9 | HPRT1 | hypoxanthine phosphoribosyltransferase 1 | 311 | 0.132 | 0.1275 | No |
| 10 | IRF8 | interferon regulatory factor 8 | 434 | 0.115 | 0.1315 | No |
| 11 | TPST1 | tyrosylprotein sulfotransferase 1 | 453 | 0.114 | 0.1407 | No |
| 12 | PROS1 | protein S (alpha) | 556 | 0.106 | 0.1449 | No |
| 13 | CYFIP2 | cytoplasmic FMR1 interacting protein 2 | 557 | 0.106 | 0.1543 | No |
| 14 | IGFBP4 | insulin like growth factor binding protein 4 | 792 | 0.093 | 0.1504 | No |
| 15 | HNF4A | hepatocyte nuclear factor 4, alpha | 1031 | 0.083 | 0.1455 | No |
| 16 | FBLN1 | fibulin 1 | 1105 | 0.081 | 0.1489 | No |
| 17 | ID2 | inhibitor of DNA binding 2, dominant negative helix-loop-helix protein | 1150 | 0.080 | 0.1537 | No |
| 18 | TDO2 | tryptophan 2,3-dioxygenase | 1202 | 0.079 | 0.1581 | No |
| 19 | F11 | coagulation factor XI | 1205 | 0.078 | 0.1650 | No |
| 20 | UPB1 | ureidopropionase, beta | 1309 | 0.076 | 0.1664 | No |
| 21 | F10 | coagulation factor X | 1340 | 0.075 | 0.1715 | No |
| 22 | TMEM97 | transmembrane protein 97 | 1371 | 0.075 | 0.1766 | No |
| 23 | ACOX2 | acyl-CoA oxidase 2, branched chain | 1687 | 0.067 | 0.1663 | No |
| 24 | SERTAD1 | SERTA domain containing 1 | 1727 | 0.066 | 0.1702 | No |
| 25 | FBP1 | fructose-1,6-bisphosphatase 1 | 1740 | 0.066 | 0.1754 | No |
| 26 | BPHL | biphenyl hydrolase-like (serine hydrolase) | 1755 | 0.066 | 0.1806 | No |
| 27 | GART | phosphoribosylglycinamide formyltransferase, phosphoribosylglycinamide synthetase, phosphoribosylaminoimidazole synthetase | 1825 | 0.064 | 0.1827 | No |
| 28 | PTGES | prostaglandin E synthase | 1996 | 0.061 | 0.1794 | No |
| 29 | LCAT | lecithin-cholesterol acyltransferase | 2096 | 0.060 | 0.1796 | No |
| 30 | FAH | fumarylacetoacetate hydrolase (fumarylacetoacetase) | 2119 | 0.060 | 0.1838 | No |
| 31 | CDA | cytidine deaminase | 2157 | 0.059 | 0.1871 | No |
| 32 | NDRG2 | NDRG family member 2 | 2169 | 0.059 | 0.1918 | No |
| 33 | NFS1 | NFS1 cysteine desulfurase | 2282 | 0.057 | 0.1911 | No |
| 34 | RBP4 | retinol binding protein 4, plasma | 2424 | 0.055 | 0.1887 | No |
| 35 | ETS2 | v-ets avian erythroblastosis virus E26 oncogene homolog 2 | 2456 | 0.054 | 0.1919 | No |
| 36 | CYP4F2 | cytochrome P450, family 4, subfamily F, polypeptide 2 | 2548 | 0.053 | 0.1919 | No |
| 37 | DHRS1 | dehydrogenase/reductase (SDR family) member 1 | 2706 | 0.051 | 0.1883 | No |
| 38 | IGFBP1 | insulin like growth factor binding protein 1 | 2762 | 0.050 | 0.1900 | No |
| 39 | CASP6 | caspase 6 | 2891 | 0.049 | 0.1877 | No |
| 40 | PTGDS | prostaglandin D2 synthase 21kDa (brain) | 2903 | 0.049 | 0.1914 | No |
| 41 | CCL25 | chemokine (C-C motif) ligand 25 | 3110 | 0.046 | 0.1849 | No |
| 42 | GSTM4 | glutathione S-transferase mu 4 | 3116 | 0.046 | 0.1888 | No |
| 43 | PGD | phosphogluconate dehydrogenase | 3163 | 0.046 | 0.1905 | No |
| 44 | MTHFD1 | methylenetetrahydrofolate dehydrogenase (NADP+ dependent) 1, methenyltetrahydrofolate cyclohydrolase, formyltetrahydrofolate synthetase | 3385 | 0.044 | 0.1830 | No |
| 45 | ENTPD5 | ectonucleoside triphosphate diphosphohydrolase 5 | 3473 | 0.043 | 0.1823 | No |
| 46 | HMOX1 | heme oxygenase 1 | 3502 | 0.043 | 0.1846 | No |
| 47 | TGFB2 | transforming growth factor beta 2 | 3684 | 0.041 | 0.1789 | No |
| 48 | VTN | vitronectin | 3798 | 0.040 | 0.1766 | No |
| 49 | PINK1 | PTEN induced putative kinase 1 | 3839 | 0.039 | 0.1780 | No |
| 50 | CYP2J2 | cytochrome P450, family 2, subfamily J, polypeptide 2 | 3868 | 0.039 | 0.1800 | No |
| 51 | DHRS7 | dehydrogenase/reductase (SDR family) member 7 | 3908 | 0.038 | 0.1814 | No |
| 52 | SLC35D1 | solute carrier family 35 (UDP-GlcA/UDP-GalNAc transporter), member D1 | 3925 | 0.038 | 0.1840 | No |
| 53 | PTGES3 | prostaglandin E synthase 3 (cytosolic) | 4011 | 0.037 | 0.1829 | No |
| 54 | EPHA2 | EPH receptor A2 | 4100 | 0.037 | 0.1816 | No |
| 55 | PLG | plasminogen | 4359 | 0.034 | 0.1713 | No |
| 56 | ITIH1 | inter-alpha-trypsin inhibitor heavy chain 1 | 4587 | 0.032 | 0.1625 | No |
| 57 | ASL | argininosuccinate lyase | 4722 | 0.031 | 0.1583 | No |
| 58 | PMM1 | phosphomannomutase 1 | 4930 | 0.030 | 0.1503 | No |
| 59 | BCAR1 | breast cancer anti-estrogen resistance 1 | 4960 | 0.030 | 0.1514 | No |
| 60 | ABCD2 | Transcript Identified by AceView, Entrez Gene ID(s) 225 | 5228 | 0.028 | 0.1400 | No |
| 61 | GSTT2 | glutathione S-transferase theta 2 (gene/pseudogene) | 5299 | 0.027 | 0.1388 | No |
| 62 | DCXR | dicarbonyl/L-xylulose reductase | 5337 | 0.027 | 0.1393 | No |
| 63 | G6PC | glucose-6-phosphatase, catalytic subunit | 5451 | 0.026 | 0.1357 | No |
| 64 | ALDH9A1 | aldehyde dehydrogenase 9 family, member A1 | 5592 | 0.025 | 0.1307 | No |
| 65 | CYP1A2 | cytochrome P450, family 1, subfamily A, polypeptide 2 | 5608 | 0.025 | 0.1321 | No |
| 66 | MBL2 | mannose-binding lectin (protein C) 2, soluble | 5624 | 0.025 | 0.1335 | No |
| 67 | LEAP2 | liver expressed antimicrobial peptide 2 | 5720 | 0.024 | 0.1307 | No |
| 68 | ELOVL5 | ELOVL fatty acid elongase 5 | 5798 | 0.023 | 0.1288 | No |
| 69 | BLVRB | biliverdin reductase B | 5918 | 0.023 | 0.1247 | No |
| 70 | MCCC2 | methylcrotonoyl-CoA carboxylase 2 | 6016 | 0.022 | 0.1216 | No |
| 71 | SLC6A12 | solute carrier family 6 (neurotransmitter transporter), member 12 | 6101 | 0.021 | 0.1192 | No |
| 72 | MT2A | metallothionein 2A | 6247 | 0.021 | 0.1135 | No |
| 73 | TKFC | triokinase/FMN cyclase | 6271 | 0.020 | 0.1141 | No |
| 74 | ECH1 | enoyl-CoA hydratase 1, peroxisomal | 6583 | 0.018 | 0.0997 | No |
| 75 | HGFAC | HGF activator | 6717 | 0.018 | 0.0944 | No |
| 76 | PDLIM5 | PDZ and LIM domain 5 | 6773 | 0.017 | 0.0931 | No |
| 77 | CYP1A1 | cytochrome P450, family 1, subfamily A, polypeptide 1 | 6941 | 0.016 | 0.0859 | No |
| 78 | TNFRSF1A | tumor necrosis factor receptor superfamily, member 1A | 7027 | 0.016 | 0.0829 | No |
| 79 | GSS | glutathione synthetase | 7092 | 0.015 | 0.0809 | No |
| 80 | APOE | apolipoprotein E | 7256 | 0.014 | 0.0738 | No |
| 81 | ALDH2 | aldehyde dehydrogenase 2 family (mitochondrial) | 7424 | 0.013 | 0.0663 | No |
| 82 | UGDH | UDP-glucose 6-dehydrogenase | 7487 | 0.013 | 0.0643 | No |
| 83 | CYP27A1 | cytochrome P450, family 27, subfamily A, polypeptide 1 | 7603 | 0.012 | 0.0594 | No |
| 84 | ACOX1 | acyl-CoA oxidase 1, palmitoyl | 7694 | 0.012 | 0.0558 | No |
| 85 | CSAD | cysteine sulfinic acid decarboxylase | 7833 | 0.011 | 0.0496 | No |
| 86 | ACSM1 | acyl-CoA synthetase medium-chain family member 1 | 7846 | 0.011 | 0.0500 | No |
| 87 | PGRMC1 | progesterone receptor membrane component 1 | 7948 | 0.010 | 0.0456 | No |
| 88 | PTGR1 | prostaglandin reductase 1 | 8064 | 0.009 | 0.0405 | No |
| 89 | PC | pyruvate carboxylase | 8281 | 0.008 | 0.0301 | No |
| 90 | GNMT | glycine N-methyltransferase | 8445 | 0.007 | 0.0223 | No |
| 91 | PEMT | phosphatidylethanolamine N-methyltransferase | 8772 | 0.005 | 0.0059 | No |
| 92 | HRG | histidine-rich glycoprotein | 8796 | 0.005 | 0.0052 | No |
| 93 | AHCY | adenosylhomocysteinase | 8841 | 0.005 | 0.0033 | No |
| 94 | SLC6A6 | solute carrier family 6 (neurotransmitter transporter), member 6 | 9077 | 0.004 | -0.0085 | No |
| 95 | FMO3 | flavin containing monooxygenase 3 | 9353 | 0.002 | -0.0225 | No |
| 96 | PPARD | peroxisome proliferator-activated receptor delta | 9547 | 0.001 | -0.0324 | No |
| 97 | KYNU | kynureninase | 9602 | 0.001 | -0.0351 | No |
| 98 | NQO1 | NAD(P)H dehydrogenase, quinone 1 | 9643 | 0.001 | -0.0371 | No |
| 99 | SPINT2 | serine peptidase inhibitor, Kunitz type, 2 | 9802 | -0.000 | -0.0453 | No |
| 100 | GSR | glutathione reductase | 9874 | -0.000 | -0.0489 | No |
| 101 | ITIH4 | inter-alpha-trypsin inhibitor heavy chain family, member 4 | 9890 | -0.001 | -0.0496 | No |
| 102 | HES6 | hes family bHLH transcription factor 6 | 9921 | -0.001 | -0.0511 | No |
| 103 | NMT1 | N-myristoyltransferase 1 | 10101 | -0.002 | -0.0602 | No |
| 104 | SLC12A4 | solute carrier family 12 (potassium/chloride transporter), member 4 | 10253 | -0.003 | -0.0678 | No |
| 105 | GCKR | glucokinase (hexokinase 4) regulator | 10274 | -0.003 | -0.0686 | No |
| 106 | CRP | C-reactive protein, pentraxin-related | 10499 | -0.004 | -0.0798 | No |
| 107 | ACO2 | aconitase 2, mitochondrial | 10537 | -0.004 | -0.0814 | No |
| 108 | SERPINA6 | serpin peptidase inhibitor, clade A (alpha-1 antiproteinase, antitrypsin), member 6 | 10647 | -0.005 | -0.0865 | No |
| 109 | ARG1 | arginase 1 | 10703 | -0.005 | -0.0889 | No |
| 110 | ALAS1 | 5-aminolevulinate synthase 1 | 10706 | -0.005 | -0.0885 | No |
| 111 | GSTA3 | glutathione S-transferase alpha 3 | 10775 | -0.006 | -0.0915 | No |
| 112 | NINJ1 | ninjurin 1 | 10805 | -0.006 | -0.0925 | No |
| 113 | AP4B1 | adaptor-related protein complex 4, beta 1 subunit | 10843 | -0.006 | -0.0939 | No |
| 114 | DDT | D-dopachrome tautomerase | 10925 | -0.007 | -0.0975 | No |
| 115 | COMT | catechol-O-methyltransferase | 11171 | -0.008 | -0.1094 | No |
| 116 | CNDP2 | CNDP dipeptidase 2 (metallopeptidase M20 family) | 11311 | -0.009 | -0.1158 | No |
| 117 | HACL1 | 2-hydroxyacyl-CoA lyase 1 | 11435 | -0.010 | -0.1213 | No |
| 118 | DHPS | deoxyhypusine synthase | 11436 | -0.010 | -0.1205 | No |
| 119 | ATP2A2 | ATPase, Ca++ transporting, cardiac muscle, slow twitch 2 | 11662 | -0.011 | -0.1312 | No |
| 120 | ABHD6 | abhydrolase domain containing 6 | 11856 | -0.012 | -0.1401 | No |
| 121 | ATOH8 | atonal bHLH transcription factor 8 | 12057 | -0.013 | -0.1493 | No |
| 122 | TMBIM6 | transmembrane BAX inhibitor motif containing 6 | 12126 | -0.014 | -0.1516 | No |
| 123 | SMOX | spermine oxidase | 12281 | -0.015 | -0.1582 | No |
| 124 | CAT | catalase | 12431 | -0.016 | -0.1646 | No |
| 125 | AOX1 | aldehyde oxidase 1 | 12435 | -0.016 | -0.1633 | No |
| 126 | ETFDH | electron-transferring-flavoprotein dehydrogenase | 12478 | -0.016 | -0.1641 | No |
| 127 | CBR1 | carbonyl reductase 1 | 12637 | -0.017 | -0.1707 | No |
| 128 | SAR1B | Transcript Identified by AceView, Entrez Gene ID(s) 51128 | 12728 | -0.017 | -0.1738 | No |
| 129 | GCH1 | GTP cyclohydrolase 1 | 12793 | -0.018 | -0.1756 | No |
| 130 | ABCC3 | ATP binding cassette subfamily C member 3 | 12874 | -0.018 | -0.1781 | No |
| 131 | EPHX1 | epoxide hydrolase 1, microsomal (xenobiotic) | 12904 | -0.019 | -0.1779 | No |
| 132 | SERPINE1 | serpin peptidase inhibitor, clade E (nexin, plasminogen activator inhibitor type 1), member 1 | 12974 | -0.019 | -0.1798 | No |
| 133 | TAT | tyrosine aminotransferase | 13028 | -0.019 | -0.1808 | No |
| 134 | CYP2S1 | cytochrome P450, family 2, subfamily S, polypeptide 1 | 13179 | -0.020 | -0.1867 | No |
| 135 | JUP | junction plakoglobin | 13218 | -0.021 | -0.1869 | No |
| 136 | CD36 | CD36 molecule (thrombospondin receptor) | 13245 | -0.021 | -0.1864 | No |
| 137 | GAD1 | glutamate decarboxylase 1 | 13246 | -0.021 | -0.1845 | No |
| 138 | CYB5A | cytochrome b5 type A (microsomal) | 13471 | -0.022 | -0.1941 | No |
| 139 | ANGPTL3 | angiopoietin like 3 | 13931 | -0.026 | -0.2155 | No |
| 140 | CYP17A1 | cytochrome P450, family 17, subfamily A, polypeptide 1 | 13974 | -0.026 | -0.2154 | No |
| 141 | SLC35B1 | solute carrier family 35, member B1 | 14029 | -0.026 | -0.2158 | No |
| 142 | LONP1 | lon peptidase 1, mitochondrial | 14072 | -0.027 | -0.2156 | No |
| 143 | ARPP19 | cAMP-regulated phosphoprotein 19kDa | 14107 | -0.027 | -0.2150 | No |
| 144 | PTS | 6-pyruvoyltetrahydropterin synthase | 14234 | -0.028 | -0.2190 | No |
| 145 | ACP1 | acid phosphatase 1, soluble | 14417 | -0.030 | -0.2257 | No |
| 146 | ADH5 | alcohol dehydrogenase 5 (class III), chi polypeptide | 14447 | -0.030 | -0.2246 | No |
| 147 | ADH1C | alcohol dehydrogenase 1C (class I), gamma polypeptide | 14631 | -0.031 | -0.2312 | No |
| 148 | HSD11B1 | hydroxysteroid (11-beta) dehydrogenase 1 | 14833 | -0.033 | -0.2387 | No |
| 149 | IL1R1 | interleukin 1 receptor, type I | 14863 | -0.033 | -0.2372 | No |
| 150 | SLC22A1 | solute carrier family 22 (organic cation transporter), member 1 | 14949 | -0.034 | -0.2386 | No |
| 151 | IDH1 | isocitrate dehydrogenase 1 (NADP+) | 14950 | -0.034 | -0.2356 | No |
| 152 | SHMT2 | serine hydroxymethyltransferase 2 (mitochondrial) | 14979 | -0.034 | -0.2340 | No |
| 153 | REG1A | regenerating islet-derived 1 alpha | 15058 | -0.035 | -0.2349 | No |
| 154 | MPP2 | membrane protein, palmitoylated 2 | 15082 | -0.035 | -0.2329 | No |
| 155 | PYCR1 | pyrroline-5-carboxylate reductase 1 | 15126 | -0.036 | -0.2320 | No |
| 156 | PSMB10 | proteasome subunit beta 10 | 15143 | -0.036 | -0.2297 | No |
| 157 | SSR3 | signal sequence receptor, gamma (translocon-associated protein gamma) | 15803 | -0.042 | -0.2600 | No |
| 158 | ARG2 | arginase 2 | 16201 | -0.046 | -0.2765 | No |
| 159 | DDAH2 | dimethylarginine dimethylaminohydrolase 2 | 16235 | -0.046 | -0.2741 | No |
| 160 | ACOX3 | acyl-CoA oxidase 3, pristanoyl | 16291 | -0.047 | -0.2727 | No |
| 161 | FMO1 | flavin containing monooxygenase 1 | 16324 | -0.047 | -0.2702 | No |
| 162 | NPC1 | Niemann-Pick disease, type C1 | 16604 | -0.051 | -0.2800 | No |
| 163 | ADH7 | alcohol dehydrogenase 7 (class IV), mu or sigma polypeptide | 16808 | -0.054 | -0.2857 | No |
| 164 | PDK4 | pyruvate dehydrogenase kinase, isozyme 4 | 16941 | -0.056 | -0.2876 | No |
| 165 | IGF1 | insulin-like growth factor 1 (somatomedin C) | 17090 | -0.058 | -0.2900 | Yes |
| 166 | BCAT1 | branched chain amino-acid transaminase 1, cytosolic | 17138 | -0.059 | -0.2872 | Yes |
| 167 | LPIN2 | lipin 2 | 17229 | -0.060 | -0.2865 | Yes |
| 168 | CYP26A1 | cytochrome P450, family 26, subfamily A, polypeptide 1 | 17252 | -0.061 | -0.2822 | Yes |
| 169 | FETUB | fetuin B | 17269 | -0.061 | -0.2776 | Yes |
| 170 | ESR1 | estrogen receptor 1 | 17484 | -0.065 | -0.2829 | Yes |
| 171 | SLC46A3 | solute carrier family 46, member 3 | 17585 | -0.067 | -0.2821 | Yes |
| 172 | VNN1 | vanin 1 | 17657 | -0.069 | -0.2797 | Yes |
| 173 | RETSAT | retinol saturase (all-trans-retinol 13,14-reductase) | 17683 | -0.069 | -0.2748 | Yes |
| 174 | GCLC | glutamate-cysteine ligase, catalytic subunit | 17981 | -0.077 | -0.2834 | Yes |
| 175 | CDO1 | cysteine dioxygenase type 1 | 18137 | -0.081 | -0.2842 | Yes |
| 176 | ALDH3A1 | aldehyde dehydrogenase 3 family, member A1 | 18146 | -0.081 | -0.2774 | Yes |
| 177 | PAPSS2 | 3-phosphoadenosine 5-phosphosulfate synthase 2 | 18188 | -0.082 | -0.2722 | Yes |
| 178 | AQP9 | aquaporin 9 | 18219 | -0.083 | -0.2663 | Yes |
| 179 | UPP1 | uridine phosphorylase 1 | 18404 | -0.089 | -0.2679 | Yes |
| 180 | SLC1A5 | solute carrier family 1 (neutral amino acid transporter), member 5 | 18428 | -0.090 | -0.2611 | Yes |
| 181 | TMEM176B | transmembrane protein 176B | 18831 | -0.112 | -0.2720 | Yes |
| 182 | CYP2C18 | cytochrome P450, family 2, subfamily C, polypeptide 18 | 18918 | -0.119 | -0.2658 | Yes |
| 183 | POR | P450 (cytochrome) oxidoreductase | 18989 | -0.126 | -0.2583 | Yes |
| 184 | ACP2 | acid phosphatase 2, lysosomal | 19128 | -0.146 | -0.2524 | Yes |
| 185 | MAOA | monoamine oxidase A | 19140 | -0.148 | -0.2398 | Yes |
| 186 | ABCC2 | ATP binding cassette subfamily C member 2 | 19152 | -0.150 | -0.2270 | Yes |
| 187 | DDC | dopa decarboxylase | 19169 | -0.153 | -0.2142 | Yes |
| 188 | GABARAPL1 | GABA(A) receptor-associated protein like 1 | 19244 | -0.172 | -0.2027 | Yes |
| 189 | ENPEP | glutamyl aminopeptidase (aminopeptidase A) | 19267 | -0.180 | -0.1879 | Yes |
| 190 | RAP1GAP | RAP1 GTPase activating protein | 19313 | -0.196 | -0.1727 | Yes |
| 191 | XDH | xanthine dehydrogenase | 19320 | -0.197 | -0.1554 | Yes |
| 192 | CES1 | carboxylesterase 1 | 19333 | -0.202 | -0.1381 | Yes |
| 193 | TYR | tyrosinase | 19357 | -0.216 | -0.1200 | Yes |
| 194 | CROT | carnitine O-octanoyltransferase | 19375 | -0.223 | -0.1010 | Yes |
| 195 | MAN1A1 | mannosidase, alpha, class 1A, member 1 | 19386 | -0.229 | -0.0811 | Yes |
| 196 | CYP2E1 | cytochrome P450, family 2, subfamily E, polypeptide 1 | 19389 | -0.233 | -0.0605 | Yes |
| 197 | AKR1C3 | aldo-keto reductase family 1, member C3 | 19455 | -0.304 | -0.0368 | Yes |
| 198 | AKR1C2 | aldo-keto reductase family 1, member C2 | 19512 | -0.452 | 0.0006 | Yes |
Table: GSEA details [plain text format]

  

Fig 2: HALLMARK\_XENOBIOTIC\_METABOLISM      
 Blue-Pink O' Gram in the Space of the Analyzed GeneSet

  

Fig 3: HALLMARK\_XENOBIOTIC\_METABOLISM: Random ES distribution      
 Gene set null distribution of ES for **HALLMARK\_XENOBIOTIC\_METABOLISM**
