# Supplementary material for: Somatic Hypomethylation of Pericentromeric SST1 Repeats and Tetraploidization in Human Colorectal Cancer Cells
Source: Cancers (Basel). 2021 Oct 26;13(21):5353. doi: 10.3390/cancers13215353 (PMC8582499; doi:10.3390/cancers13215353)
Supplement: Supplementary file 1 [file cancers-13-05353-s001.zip › cancers-1346744-supplementary-author confirmation-/GSEA.zip!/GSEA_TETRvsDIP/HALLMARK_HYPOXIA.html]

Details for gene set HALLMARK\_HYPOXIA[GSEA]

|  || Dataset | eset\_byprobe\_collapsed\_to\_symbols.Diploid\_vs\_Tetraploid.cls #Tetraploid\_versus\_Diploid.Diploid\_vs\_Tetraploid.cls #Tetraploid\_versus\_Diploid\_repos |
| Phenotype | Diploid\_vs\_Tetraploid.cls#Tetraploid\_versus\_Diploid\_repos |
| Upregulated in class | Diploid |
| GeneSet | HALLMARK\_HYPOXIA |
| Enrichment Score (ES) | -0.30255848 |
| Normalized Enrichment Score (NES) | -1.2446362 |
| Nominal p-value | 0.082212254 |
| FDR q-value | 0.30118522 |
| FWER p-Value | 0.965 |
Table: GSEA Results Summary

  

Fig 1: Enrichment plot: HALLMARK\_HYPOXIA      
 Profile of the Running ES Score & Positions of GeneSet Members on the Rank Ordered List

  

| SYMBOL | TITLE | RANK IN GENE LIST | RANK METRIC SCORE | RUNNING ES | CORE ENRICHMENT || 1 | S100A4 | S100 calcium binding protein A4 | 5 | 0.471 | 0.0394 | No |
| 2 | PLAC8 | placenta specific 8 | 13 | 0.403 | 0.0730 | No |
| 3 | CHST3 | carbohydrate (chondroitin 6) sulfotransferase 3 | 46 | 0.291 | 0.0959 | No |
| 4 | RRAGD | Ras-related GTP binding D | 54 | 0.280 | 0.1191 | No |
| 5 | GCNT2 | glucosaminyl (N-acetyl) transferase 2, I-branching enzyme (I blood group) | 76 | 0.233 | 0.1376 | No |
| 6 | F3 | coagulation factor III (thromboplastin, tissue factor) | 119 | 0.191 | 0.1515 | No |
| 7 | ALDOC | aldolase C, fructose-bisphosphate | 407 | 0.118 | 0.1466 | No |
| 8 | EFNA3 | ephrin-A3 | 448 | 0.114 | 0.1542 | No |
| 9 | ACKR3 | atypical chemokine receptor 3 | 455 | 0.114 | 0.1634 | No |
| 10 | SELENBP1 | selenium binding protein 1 | 563 | 0.105 | 0.1667 | No |
| 11 | IGFBP3 | insulin like growth factor binding protein 3 | 582 | 0.104 | 0.1746 | No |
| 12 | ADORA2B | adenosine A2b receptor | 596 | 0.104 | 0.1826 | No |
| 13 | CDKN1A | cyclin-dependent kinase inhibitor 1A (p21, Cip1) | 690 | 0.097 | 0.1860 | No |
| 14 | TGM2 | transglutaminase 2 | 714 | 0.096 | 0.1929 | No |
| 15 | KIF5A | kinesin family member 5A | 840 | 0.091 | 0.1941 | No |
| 16 | IRS2 | insulin receptor substrate 2 | 1045 | 0.083 | 0.1905 | No |
| 17 | TGFBI | transforming growth factor, beta-induced, 68kDa | 1130 | 0.080 | 0.1929 | No |
| 18 | DPYSL4 | dihydropyrimidinase-like 4 | 1272 | 0.077 | 0.1921 | No |
| 19 | TPST2 | tyrosylprotein sulfotransferase 2 | 1275 | 0.077 | 0.1985 | No |
| 20 | NDST2 | N-deacetylase/N-sulfotransferase (heparan glucosaminyl) 2 | 1343 | 0.075 | 0.2014 | No |
| 21 | PLAUR | plasminogen activator, urokinase receptor | 1355 | 0.075 | 0.2071 | No |
| 22 | KLF7 | Kruppel-like factor 7 (ubiquitous) | 1362 | 0.075 | 0.2131 | No |
| 23 | EGFR | epidermal growth factor receptor | 1396 | 0.074 | 0.2176 | No |
| 24 | SLC25A1 | solute carrier family 25 (mitochondrial carrier; citrate transporter), member 1 | 1475 | 0.072 | 0.2196 | No |
| 25 | PGAM2 | phosphoglycerate mutase 2 (muscle) | 1477 | 0.072 | 0.2257 | No |
| 26 | FBP1 | fructose-1,6-bisphosphatase 1 | 1740 | 0.066 | 0.2177 | No |
| 27 | ANGPTL4 | angiopoietin like 4 | 1873 | 0.064 | 0.2162 | No |
| 28 | PCK1 | phosphoenolpyruvate carboxykinase 1 (soluble) | 1909 | 0.063 | 0.2197 | No |
| 29 | HS3ST1 | heparan sulfate (glucosamine) 3-O-sulfotransferase 1 | 2068 | 0.060 | 0.2166 | No |
| 30 | NCAN | neurocan | 2359 | 0.056 | 0.2063 | No |
| 31 | CA12 | carbonic anhydrase XII | 2439 | 0.055 | 0.2068 | No |
| 32 | SDC2 | syndecan 2 | 2442 | 0.055 | 0.2113 | No |
| 33 | ANXA2 | annexin A2 | 2591 | 0.053 | 0.2081 | No |
| 34 | UGP2 | UDP-glucose pyrophosphorylase 2 | 2703 | 0.051 | 0.2067 | No |
| 35 | IGFBP1 | insulin like growth factor binding protein 1 | 2762 | 0.050 | 0.2079 | No |
| 36 | CSRP2 | cysteine and glycine-rich protein 2 | 2804 | 0.050 | 0.2100 | No |
| 37 | CASP6 | caspase 6 | 2891 | 0.049 | 0.2096 | No |
| 38 | EDN2 | endothelin 2 | 2912 | 0.048 | 0.2127 | No |
| 39 | MAFF | v-maf avian musculoaponeurotic fibrosarcoma oncogene homolog F | 3075 | 0.047 | 0.2082 | No |
| 40 | ENO2 | enolase 2 (gamma, neuronal) | 3086 | 0.047 | 0.2117 | No |
| 41 | STC1 | stanniocalcin 1 | 3174 | 0.046 | 0.2110 | No |
| 42 | HMOX1 | heme oxygenase 1 | 3502 | 0.043 | 0.1977 | No |
| 43 | MYH9 | Jeck2013 ANTISENSE, CDS, coding, INTERNAL, intronic, OVCODE, OVEXON best transcript NM\_002473 | 3583 | 0.042 | 0.1971 | No |
| 44 | AMPD3 | adenosine monophosphate deaminase 3 | 3847 | 0.039 | 0.1867 | No |
| 45 | SLC37A4 | solute carrier family 37 (glucose-6-phosphate transporter), member 4 | 3916 | 0.038 | 0.1865 | No |
| 46 | NR3C1 | nuclear receptor subfamily 3, group C, member 1 (glucocorticoid receptor) | 4470 | 0.033 | 0.1607 | No |
| 47 | PFKFB3 | 6-phosphofructo-2-kinase/fructose-2,6-biphosphatase 3 | 4583 | 0.032 | 0.1576 | No |
| 48 | TPI1 | triosephosphate isomerase 1 | 4710 | 0.031 | 0.1537 | No |
| 49 | MIF | macrophage migration inhibitory factor (glycosylation-inhibiting factor) | 4925 | 0.030 | 0.1452 | No |
| 50 | INHA | inhibin alpha | 5003 | 0.029 | 0.1437 | No |
| 51 | DTNA | dystrobrevin, alpha | 5229 | 0.028 | 0.1344 | No |
| 52 | JMJD6 | jumonji domain containing 6 | 5524 | 0.025 | 0.1213 | No |
| 53 | VLDLR | very low density lipoprotein receptor | 5549 | 0.025 | 0.1221 | No |
| 54 | ANKZF1 | ankyrin repeat and zinc finger domain containing 1 | 5647 | 0.024 | 0.1192 | No |
| 55 | GPI | glucose-6-phosphate isomerase | 5659 | 0.024 | 0.1207 | No |
| 56 | LDHA | lactate dehydrogenase A | 6019 | 0.022 | 0.1039 | No |
| 57 | CHST2 | carbohydrate (N-acetylglucosamine-6-O) sulfotransferase 2 | 6022 | 0.022 | 0.1057 | No |
| 58 | MT1E | metallothionein 1E | 6127 | 0.021 | 0.1021 | No |
| 59 | B4GALNT2 | beta-1,4-N-acetyl-galactosaminyl transferase 2 | 6135 | 0.021 | 0.1035 | No |
| 60 | MT2A | metallothionein 2A | 6247 | 0.021 | 0.0995 | No |
| 61 | B3GALT6 | UDP-Gal:betaGal beta 1,3-galactosyltransferase 6 | 6311 | 0.020 | 0.0979 | No |
| 62 | ALDOA | aldolase A, fructose-bisphosphate | 6361 | 0.020 | 0.0971 | No |
| 63 | MXI1 | MAX interactor 1, dimerization protein | 6513 | 0.019 | 0.0909 | No |
| 64 | CP | ceruloplasmin (ferroxidase) | 6778 | 0.017 | 0.0787 | No |
| 65 | PPP1R3C | protein phosphatase 1, regulatory subunit 3C | 6836 | 0.017 | 0.0771 | No |
| 66 | MAP3K1 | mitogen-activated protein kinase kinase kinase 1, E3 ubiquitin protein ligase | 7078 | 0.015 | 0.0659 | No |
| 67 | HAS1 | hyaluronan synthase 1 | 7235 | 0.015 | 0.0591 | No |
| 68 | LXN | latexin | 7345 | 0.014 | 0.0546 | No |
| 69 | WSB1 | WD repeat and SOCS box containing 1 | 7572 | 0.013 | 0.0440 | No |
| 70 | GAPDHS | glyceraldehyde-3-phosphate dehydrogenase, spermatogenic | 7644 | 0.012 | 0.0413 | No |
| 71 | FOSL2 | FOS-like antigen 2 | 7696 | 0.012 | 0.0397 | No |
| 72 | BTG1 | B-cell translocation gene 1, anti-proliferative | 7702 | 0.012 | 0.0404 | No |
| 73 | NAGK | N-acetylglucosamine kinase | 7952 | 0.010 | 0.0284 | No |
| 74 | NDST1 | N-deacetylase/N-sulfotransferase (heparan glucosaminyl) 1 | 7971 | 0.010 | 0.0283 | No |
| 75 | FOXO3 | forkhead box O3 | 8135 | 0.009 | 0.0206 | No |
| 76 | ILVBL | ilvB (bacterial acetolactate synthase)-like | 8150 | 0.009 | 0.0207 | No |
| 77 | PFKP | phosphofructokinase, platelet | 8326 | 0.008 | 0.0123 | No |
| 78 | LDHC | lactate dehydrogenase C | 8359 | 0.008 | 0.0113 | No |
| 79 | GRHPR | glyoxylate reductase/hydroxypyruvate reductase | 8421 | 0.007 | 0.0087 | No |
| 80 | SAP30 | Sin3A associated protein 30kDa | 8495 | 0.007 | 0.0055 | No |
| 81 | HK2 | hexokinase 2 | 8575 | 0.006 | 0.0020 | No |
| 82 | BNIP3L | BCL2/adenovirus E1B 19kDa interacting protein 3-like | 8648 | 0.006 | -0.0012 | No |
| 83 | ALDOB | aldolase B, fructose-bisphosphate | 8657 | 0.006 | -0.0011 | No |
| 84 | PFKL | phosphofructokinase, liver | 8698 | 0.006 | -0.0027 | No |
| 85 | CXCR4 | chemokine (C-X-C motif) receptor 4 | 8789 | 0.005 | -0.0069 | No |
| 86 | SCARB1 | scavenger receptor class B, member 1 | 8987 | 0.004 | -0.0168 | No |
| 87 | PLIN2 | perilipin 2 | 9014 | 0.004 | -0.0178 | No |
| 88 | SLC6A6 | solute carrier family 6 (neurotransmitter transporter), member 6 | 9077 | 0.004 | -0.0207 | No |
| 89 | NDRG1 | N-myc downstream regulated 1 | 9078 | 0.004 | -0.0204 | No |
| 90 | TPD52 | tumor protein D52 | 9288 | 0.003 | -0.0310 | No |
| 91 | XPNPEP1 | X-prolyl aminopeptidase (aminopeptidase P) 1, soluble | 9532 | 0.001 | -0.0435 | No |
| 92 | TGFB3 | transforming growth factor beta 3 | 9685 | 0.001 | -0.0513 | No |
| 93 | CCNG2 | cyclin G2 | 9692 | 0.000 | -0.0515 | No |
| 94 | GAPDH | glyceraldehyde-3-phosphate dehydrogenase | 9830 | -0.000 | -0.0586 | No |
| 95 | HK1 | hexokinase 1 | 10171 | -0.002 | -0.0760 | No |
| 96 | ENO3 | enolase 3 (beta, muscle) | 10237 | -0.003 | -0.0792 | No |
| 97 | PGM2 | phosphoglucomutase 2 | 10296 | -0.003 | -0.0819 | No |
| 98 | ERRFI1 | ERBB receptor feedback inhibitor 1 | 10474 | -0.004 | -0.0907 | No |
| 99 | GAA | glucosidase, alpha; acid | 10525 | -0.004 | -0.0930 | No |
| 100 | PGF | placental growth factor | 10639 | -0.005 | -0.0984 | No |
| 101 | LALBA | lactalbumin, alpha- | 10658 | -0.005 | -0.0989 | No |
| 102 | FAM162A | family with sequence similarity 162, member A | 10663 | -0.005 | -0.0987 | No |
| 103 | TIPARP | TCDD-inducible poly(ADP-ribose) polymerase | 11003 | -0.007 | -0.1156 | No |
| 104 | PGK1 | phosphoglycerate kinase 1 | 11128 | -0.008 | -0.1214 | No |
| 105 | SLC2A1 | solute carrier family 2 (facilitated glucose transporter), member 1 | 11178 | -0.008 | -0.1232 | No |
| 106 | CDKN1C | cyclin-dependent kinase inhibitor 1C (p57, Kip2) | 11246 | -0.008 | -0.1259 | No |
| 107 | PIM1 | Pim-1 proto-oncogene, serine/threonine kinase | 11480 | -0.010 | -0.1372 | No |
| 108 | GPC1 | glypican 1 | 11601 | -0.010 | -0.1425 | No |
| 109 | ENO1 | Memczak2013 ANTISENSE, coding, INTERNAL, UTR3 best transcript NM\_001428 | 11613 | -0.010 | -0.1422 | No |
| 110 | IL6 | interleukin 6 | 11825 | -0.012 | -0.1521 | No |
| 111 | HEXA | hexosaminidase A (alpha polypeptide) | 12762 | -0.018 | -0.1990 | No |
| 112 | KLF6 | Kruppel-like factor 6 | 12945 | -0.019 | -0.2069 | No |
| 113 | SERPINE1 | serpin peptidase inhibitor, clade E (nexin, plasminogen activator inhibitor type 1), member 1 | 12974 | -0.019 | -0.2067 | No |
| 114 | BRS3 | bombesin-like receptor 3 | 13005 | -0.019 | -0.2066 | No |
| 115 | TNFAIP3 | tumor necrosis factor, alpha-induced protein 3 | 13363 | -0.022 | -0.2233 | No |
| 116 | SIAH2 | siah E3 ubiquitin protein ligase 2 | 13372 | -0.022 | -0.2219 | No |
| 117 | BCAN | brevican | 13530 | -0.023 | -0.2281 | No |
| 118 | AKAP12 | A kinase (PRKA) anchor protein 12 | 13539 | -0.023 | -0.2266 | No |
| 119 | P4HA1 | prolyl 4-hydroxylase, alpha polypeptide I | 13604 | -0.023 | -0.2279 | No |
| 120 | PAM | peptidylglycine alpha-amidating monooxygenase | 13616 | -0.023 | -0.2265 | No |
| 121 | PRKCA | protein kinase C, alpha | 13659 | -0.024 | -0.2267 | No |
| 122 | PPARGC1A | peroxisome proliferator-activated receptor gamma, coactivator 1 alpha | 13685 | -0.024 | -0.2259 | No |
| 123 | ETS1 | v-ets avian erythroblastosis virus E26 oncogene homolog 1 | 13826 | -0.025 | -0.2311 | No |
| 124 | ERO1A | endoplasmic reticulum oxidoreductase alpha | 13862 | -0.025 | -0.2307 | No |
| 125 | IER3 | immediate early response 3 | 14076 | -0.027 | -0.2395 | No |
| 126 | GYS1 | glycogen synthase 1 (muscle) | 14325 | -0.029 | -0.2499 | No |
| 127 | BCL2 | B-cell CLL/lymphoma 2 | 14503 | -0.030 | -0.2565 | No |
| 128 | VHL | von Hippel-Lindau tumor suppressor, E3 ubiquitin protein ligase | 14584 | -0.031 | -0.2580 | No |
| 129 | RBPJ | recombination signal binding protein for immunoglobulin kappa J region | 14632 | -0.031 | -0.2578 | No |
| 130 | GALK1 | galactokinase 1 | 14731 | -0.032 | -0.2601 | No |
| 131 | PPFIA4 | protein tyrosine phosphatase, receptor type, f polypeptide (PTPRF), interacting protein (liprin), alpha 4 | 14744 | -0.032 | -0.2580 | No |
| 132 | DCN | decorin | 15284 | -0.037 | -0.2828 | No |
| 133 | PNRC1 | proline-rich nuclear receptor coactivator 1 | 15332 | -0.037 | -0.2821 | No |
| 134 | SRPX | sushi-repeat containing protein, X-linked | 15588 | -0.040 | -0.2920 | No |
| 135 | PKP1 | plakophilin 1 | 15607 | -0.040 | -0.2896 | No |
| 136 | PDGFB | platelet-derived growth factor beta polypeptide | 15682 | -0.041 | -0.2900 | No |
| 137 | ZNF292 | zinc finger protein 292 | 15825 | -0.042 | -0.2938 | No |
| 138 | P4HA2 | prolyl 4-hydroxylase, alpha polypeptide II | 15996 | -0.044 | -0.2989 | Yes |
| 139 | CAV1 | caveolin 1 | 16000 | -0.044 | -0.2954 | Yes |
| 140 | NEDD4L | neural precursor cell expressed, developmentally down-regulated 4-like, E3 ubiquitin protein ligase | 16038 | -0.044 | -0.2936 | Yes |
| 141 | SLC2A3 | solute carrier family 2 (facilitated glucose transporter), member 3 | 16073 | -0.045 | -0.2916 | Yes |
| 142 | NOCT | nocturnin | 16232 | -0.046 | -0.2958 | Yes |
| 143 | ISG20 | interferon stimulated exonuclease gene 20kDa | 16332 | -0.047 | -0.2970 | Yes |
| 144 | CDKN1B | cyclin-dependent kinase inhibitor 1B (p27, Kip1) | 16336 | -0.047 | -0.2931 | Yes |
| 145 | ADM | adrenomedullin | 16402 | -0.048 | -0.2924 | Yes |
| 146 | GLRX | glutaredoxin | 16461 | -0.049 | -0.2912 | Yes |
| 147 | GPC3 | glypican 3 | 16605 | -0.051 | -0.2943 | Yes |
| 148 | VEGFA | vascular endothelial growth factor A | 16659 | -0.052 | -0.2927 | Yes |
| 149 | PYGM | phosphorylase, glycogen, muscle | 16669 | -0.052 | -0.2887 | Yes |
| 150 | PRDX5 | peroxiredoxin 5 | 16745 | -0.053 | -0.2882 | Yes |
| 151 | KLHL24 | kelch-like family member 24 | 16830 | -0.054 | -0.2879 | Yes |
| 152 | AK4 | adenylate kinase 4 | 16854 | -0.055 | -0.2845 | Yes |
| 153 | GCK | glucokinase (hexokinase 4) | 16995 | -0.057 | -0.2870 | Yes |
| 154 | TPBG | trophoblast glycoprotein | 17065 | -0.058 | -0.2857 | Yes |
| 155 | PKLR | pyruvate kinase, liver and RBC | 17068 | -0.058 | -0.2809 | Yes |
| 156 | HDLBP | Jeck2013 ANTISENSE, coding, INTERNAL, intronic best transcript NM\_005336 | 17127 | -0.059 | -0.2790 | Yes |
| 157 | STC2 | stanniocalcin 2 | 17326 | -0.062 | -0.2840 | Yes |
| 158 | LOX | lysyl oxidase | 17422 | -0.064 | -0.2835 | Yes |
| 159 | SULT2B1 | sulfotransferase family 2B member 1 | 17511 | -0.066 | -0.2825 | Yes |
| 160 | FOS | FBJ murine osteosarcoma viral oncogene homolog | 17528 | -0.066 | -0.2778 | Yes |
| 161 | PHKG1 | phosphorylase kinase, gamma 1 (muscle) | 17529 | -0.066 | -0.2722 | Yes |
| 162 | DDIT4 | DNA damage inducible transcript 4 | 17557 | -0.066 | -0.2680 | Yes |
| 163 | KDELR3 | KDEL (Lys-Asp-Glu-Leu) endoplasmic reticulum protein retention receptor 3 | 17705 | -0.070 | -0.2698 | Yes |
| 164 | TKTL1 | transketolase-like 1 | 17783 | -0.072 | -0.2677 | Yes |
| 165 | GPC4 | glypican 4 | 17805 | -0.072 | -0.2627 | Yes |
| 166 | SLC2A5 | solute carrier family 2 (facilitated glucose/fructose transporter), member 5 | 17838 | -0.073 | -0.2583 | Yes |
| 167 | ZFP36 | ZFP36 ring finger protein | 17953 | -0.076 | -0.2578 | Yes |
| 168 | HOXB9 | homeobox B9 | 17965 | -0.076 | -0.2519 | Yes |
| 169 | KDM3A | lysine (K)-specific demethylase 3A | 17984 | -0.077 | -0.2464 | Yes |
| 170 | SDC4 | syndecan 4 | 18151 | -0.081 | -0.2482 | Yes |
| 171 | PDK1 | pyruvate dehydrogenase kinase, isozyme 1 | 18204 | -0.083 | -0.2439 | Yes |
| 172 | PDK3 | pyruvate dehydrogenase kinase, isozyme 3 | 18210 | -0.083 | -0.2372 | Yes |
| 173 | SDC3 | syndecan 3 | 18233 | -0.084 | -0.2313 | Yes |
| 174 | CITED2 | Cbp/p300-interacting transactivator, with Glu/Asp rich carboxy-terminal domain, 2 | 18283 | -0.085 | -0.2266 | Yes |
| 175 | HSPA5 | heat shock 70kDa protein 5 (glucose-regulated protein, 78kDa) | 18430 | -0.090 | -0.2266 | Yes |
| 176 | NFIL3 | nuclear factor, interleukin 3 regulated | 18475 | -0.092 | -0.2212 | Yes |
| 177 | EXT1 | exostosin glycosyltransferase 1 | 18542 | -0.094 | -0.2167 | Yes |
| 178 | IDS | iduronate 2-sulfatase | 18558 | -0.095 | -0.2094 | Yes |
| 179 | EFNA1 | ephrin-A1 | 18705 | -0.103 | -0.2083 | Yes |
| 180 | PPP1R15A | protein phosphatase 1, regulatory subunit 15A | 18867 | -0.115 | -0.2070 | Yes |
| 181 | TES | testin LIM domain protein | 18892 | -0.117 | -0.1983 | Yes |
| 182 | JUN | jun proto-oncogene | 19037 | -0.132 | -0.1946 | Yes |
| 183 | GBE1 | glucan (1,4-alpha-), branching enzyme 1 | 19141 | -0.148 | -0.1875 | Yes |
| 184 | ATP7A | ATPase, Cu++ transporting, alpha polypeptide | 19148 | -0.149 | -0.1753 | Yes |
| 185 | RORA | RAR-related orphan receptor A | 19240 | -0.171 | -0.1656 | Yes |
| 186 | BHLHE40 | basic helix-loop-helix family, member e40 | 19252 | -0.175 | -0.1514 | Yes |
| 187 | DUSP1 | dual specificity phosphatase 1 | 19260 | -0.177 | -0.1368 | Yes |
| 188 | TMEM45A | transmembrane protein 45A | 19407 | -0.245 | -0.1237 | Yes |
| 189 | ATF3 | activating transcription factor 3 | 19421 | -0.254 | -0.1030 | Yes |
| 190 | PGM1 | phosphoglucomutase 1 | 19438 | -0.280 | -0.0803 | Yes |
| 191 | DDIT3 | DNA-damage-inducible transcript 3 | 19440 | -0.282 | -0.0565 | Yes |
| 192 | COL5A1 | collagen, type V, alpha 1 | 19466 | -0.322 | -0.0306 | Yes |
| 193 | BGN | biglycan | 19498 | -0.399 | 0.0013 | Yes |
Table: GSEA details [plain text format]

  

Fig 2: HALLMARK\_HYPOXIA      
 Blue-Pink O' Gram in the Space of the Analyzed GeneSet

  

Fig 3: HALLMARK\_HYPOXIA: Random ES distribution      
 Gene set null distribution of ES for **HALLMARK\_HYPOXIA**
