# Supplementary material for: Somatic Hypomethylation of Pericentromeric SST1 Repeats and Tetraploidization in Human Colorectal Cancer Cells
Source: Cancers (Basel). 2021 Oct 26;13(21):5353. doi: 10.3390/cancers13215353 (PMC8582499; doi:10.3390/cancers13215353)
Supplement: Supplementary file 1 [file cancers-13-05353-s001.zip › cancers-1346744-supplementary-author confirmation-/GSEA.zip!/GSEA_TETRvsDIP/HALLMARK_PROTEIN_SECRETION.html]

Details for gene set HALLMARK\_PROTEIN\_SECRETION[GSEA]

|  || Dataset | eset\_byprobe\_collapsed\_to\_symbols.Diploid\_vs\_Tetraploid.cls #Tetraploid\_versus\_Diploid.Diploid\_vs\_Tetraploid.cls #Tetraploid\_versus\_Diploid\_repos |
| Phenotype | Diploid\_vs\_Tetraploid.cls#Tetraploid\_versus\_Diploid\_repos |
| Upregulated in class | Diploid |
| GeneSet | HALLMARK\_PROTEIN\_SECRETION |
| Enrichment Score (ES) | -0.45791554 |
| Normalized Enrichment Score (NES) | -1.7178751 |
| Nominal p-value | 0.0 |
| FDR q-value | 0.009367336 |
| FWER p-Value | 0.026 |
Table: GSEA Results Summary

  

Fig 1: Enrichment plot: HALLMARK\_PROTEIN\_SECRETION      
 Profile of the Running ES Score & Positions of GeneSet Members on the Rank Ordered List

  

| SYMBOL | TITLE | RANK IN GENE LIST | RANK METRIC SCORE | RUNNING ES | CORE ENRICHMENT || 1 | SH3GL2 | SH3-domain GRB2-like 2 | 156 | 0.170 | 0.0271 | No |
| 2 | CAV2 | caveolin 2 | 202 | 0.154 | 0.0567 | No |
| 3 | SSPN | sarcospan | 483 | 0.112 | 0.0654 | No |
| 4 | TSPAN8 | tetraspanin 8 | 1077 | 0.081 | 0.0517 | No |
| 5 | EGFR | epidermal growth factor receptor | 1396 | 0.074 | 0.0507 | No |
| 6 | ATP6V1B1 | ATPase, H+ transporting, lysosomal 56/58kDa, V1 subunit B1 | 1823 | 0.064 | 0.0421 | No |
| 7 | GLA | galactosidase, alpha | 2101 | 0.060 | 0.0402 | No |
| 8 | OCRL | oculocerebrorenal syndrome of Lowe | 2745 | 0.051 | 0.0176 | No |
| 9 | KRT18 | keratin 18, type I | 3794 | 0.040 | -0.0281 | No |
| 10 | ERGIC3 | ERGIC and golgi 3 | 4743 | 0.031 | -0.0705 | No |
| 11 | SOD1 | superoxide dismutase 1, soluble | 4956 | 0.030 | -0.0752 | No |
| 12 | PPT1 | palmitoyl-protein thioesterase 1 | 5049 | 0.029 | -0.0740 | No |
| 13 | GNAS | GNAS complex locus | 5134 | 0.028 | -0.0724 | No |
| 14 | CLTA | clathrin, light chain A | 5300 | 0.027 | -0.0754 | No |
| 15 | ZW10 | zw10 kinetochore protein | 5810 | 0.023 | -0.0967 | No |
| 16 | ANP32E | acidic nuclear phosphoprotein 32 family member E | 6152 | 0.021 | -0.1099 | No |
| 17 | STAM | signal transducing adaptor molecule (SH3 domain and ITAM motif) 1 | 7364 | 0.014 | -0.1694 | No |
| 18 | TMX1 | thioredoxin-related transmembrane protein 1 | 7518 | 0.013 | -0.1746 | No |
| 19 | DNM1L | dynamin 1-like | 7773 | 0.011 | -0.1854 | No |
| 20 | CTSC | cathepsin C | 8167 | 0.009 | -0.2038 | No |
| 21 | COG2 | component of oligomeric golgi complex 2 | 8314 | 0.008 | -0.2096 | No |
| 22 | RER1 | retention in endoplasmic reticulum sorting receptor 1 | 8480 | 0.007 | -0.2167 | No |
| 23 | RAB2A | RAB2A, member RAS oncogene family | 8993 | 0.004 | -0.2422 | No |
| 24 | SGMS1 | sphingomyelin synthase 1 | 9023 | 0.004 | -0.2429 | No |
| 25 | TPD52 | tumor protein D52 | 9288 | 0.003 | -0.2559 | No |
| 26 | NAPG | N-ethylmaleimide-sensitive factor attachment protein, gamma | 9351 | 0.002 | -0.2587 | No |
| 27 | RAB9A | RAB9A, member RAS oncogene family | 9525 | 0.001 | -0.2673 | No |
| 28 | M6PR | mannose-6-phosphate receptor (cation dependent) | 10105 | -0.002 | -0.2967 | No |
| 29 | VAMP4 | vesicle associated membrane protein 4 | 10166 | -0.002 | -0.2994 | No |
| 30 | ADAM10 | ADAM metallopeptidase domain 10 | 10365 | -0.003 | -0.3089 | No |
| 31 | AP2S1 | adaptor-related protein complex 2 sigma 1 subunit | 10452 | -0.004 | -0.3125 | No |
| 32 | MON2 | MON2 homolog, regulator of endosome-to-Golgi trafficking | 10498 | -0.004 | -0.3140 | No |
| 33 | STX7 | syntaxin 7 | 10582 | -0.005 | -0.3173 | No |
| 34 | RPS6KA3 | ribosomal protein S6 kinase, 90kDa, polypeptide 3 | 10766 | -0.006 | -0.3255 | No |
| 35 | VAMP3 | vesicle associated membrane protein 3 | 10859 | -0.006 | -0.3290 | No |
| 36 | AP2B1 | adaptor-related protein complex 2, beta 1 subunit | 10943 | -0.007 | -0.3319 | No |
| 37 | VPS45 | vacuolar protein sorting 45 homolog (S. cerevisiae) | 11142 | -0.008 | -0.3404 | No |
| 38 | STX16 | syntaxin 16 | 11260 | -0.009 | -0.3447 | No |
| 39 | SNAP23 | synaptosome associated protein 23kDa | 11372 | -0.009 | -0.3485 | No |
| 40 | SCAMP3 | secretory carrier membrane protein 3 | 11403 | -0.009 | -0.3481 | No |
| 41 | TOM1L1 | target of myb1 like 1 membrane trafficking protein | 11893 | -0.012 | -0.3707 | No |
| 42 | ARFIP1 | ADP-ribosylation factor interacting protein 1 | 12153 | -0.014 | -0.3812 | No |
| 43 | SCAMP1 | secretory carrier membrane protein 1 | 12402 | -0.015 | -0.3908 | No |
| 44 | STX12 | syntaxin 12 | 12441 | -0.016 | -0.3895 | No |
| 45 | AP2M1 | adaptor-related protein complex 2, mu 1 subunit | 12582 | -0.017 | -0.3932 | No |
| 46 | COPE | coatomer protein complex subunit epsilon | 12743 | -0.018 | -0.3978 | No |
| 47 | AP1G1 | adaptor-related protein complex 1, gamma 1 subunit | 12753 | -0.018 | -0.3946 | No |
| 48 | VPS4B | vacuolar protein sorting 4 homolog B (S. cerevisiae) | 12780 | -0.018 | -0.3923 | No |
| 49 | CLTC | clathrin, heavy chain (Hc) | 13075 | -0.020 | -0.4034 | No |
| 50 | CD63 | CD63 molecule | 13313 | -0.021 | -0.4111 | No |
| 51 | MAPK1 | mitogen-activated protein kinase 1 | 13405 | -0.022 | -0.4113 | No |
| 52 | TSG101 | tumor susceptibility 101 | 13515 | -0.023 | -0.4122 | No |
| 53 | PAM | peptidylglycine alpha-amidating monooxygenase | 13616 | -0.023 | -0.4125 | No |
| 54 | TMED2 | transmembrane p24 trafficking protein 2 | 13702 | -0.024 | -0.4119 | No |
| 55 | CLN5 | ceroid-lipofuscinosis, neuronal 5 | 13799 | -0.025 | -0.4117 | No |
| 56 | GOLGA4 | golgin A4 | 13836 | -0.025 | -0.4083 | No |
| 57 | SNX2 | sorting nexin 2 | 13888 | -0.026 | -0.4057 | No |
| 58 | VAMP7 | vesicle associated membrane protein 7 | 14231 | -0.028 | -0.4174 | No |
| 59 | TMED10 | transmembrane p24 trafficking protein 10 | 14305 | -0.029 | -0.4153 | No |
| 60 | USO1 | USO1 vesicle transport factor | 14373 | -0.029 | -0.4126 | No |
| 61 | NAPA | N-ethylmaleimide-sensitive factor attachment protein, alpha | 14800 | -0.033 | -0.4278 | No |
| 62 | RAB5A | RAB5A, member RAS oncogene family | 15213 | -0.036 | -0.4415 | No |
| 63 | ARF1 | ADP-ribosylation factor 1 | 15527 | -0.039 | -0.4495 | Yes |
| 64 | ARFGEF1 | ADP-ribosylation factor guanine nucleotide-exchange factor 1 (brefeldin A-inhibited) | 15611 | -0.040 | -0.4455 | Yes |
| 65 | RAB14 | RAB14, member RAS oncogene family | 15768 | -0.041 | -0.4450 | Yes |
| 66 | YIPF6 | Yip1 domain family member 6 | 15848 | -0.042 | -0.4403 | Yes |
| 67 | ABCA1 | ATP binding cassette subfamily A member 1 | 15924 | -0.043 | -0.4353 | Yes |
| 68 | RAB22A | RAB22A, member RAS oncogene family | 16170 | -0.046 | -0.4385 | Yes |
| 69 | SCRN1 | secernin 1 | 16395 | -0.048 | -0.4400 | Yes |
| 70 | KIF1B | kinesin family member 1B | 16464 | -0.049 | -0.4333 | Yes |
| 71 | AP3S1 | adaptor-related protein complex 3, sigma 1 subunit | 16602 | -0.051 | -0.4297 | Yes |
| 72 | CLCN3 | chloride channel, voltage-sensitive 3 | 17046 | -0.058 | -0.4406 | Yes |
| 73 | GBF1 | golgi brefeldin A resistant guanine nucleotide exchange factor 1 | 17383 | -0.063 | -0.4449 | Yes |
| 74 | ATP1A1 | ATPase, Na+/K+ transporting, alpha 1 polypeptide | 17404 | -0.064 | -0.4328 | Yes |
| 75 | AP3B1 | adaptor-related protein complex 3, beta 1 subunit | 17443 | -0.064 | -0.4214 | Yes |
| 76 | COPB1 | coatomer protein complex subunit beta 1 | 17604 | -0.067 | -0.4157 | Yes |
| 77 | COPB2 | coatomer protein complex subunit beta 2 (beta prime) | 17673 | -0.069 | -0.4050 | Yes |
| 78 | ARFGEF2 | ADP-ribosylation factor guanine nucleotide-exchange factor 2 (brefeldin A-inhibited) | 17674 | -0.069 | -0.3907 | Yes |
| 79 | LAMP2 | lysosomal-associated membrane protein 2 | 17747 | -0.071 | -0.3798 | Yes |
| 80 | IGF2R | insulin-like growth factor 2 receptor | 17794 | -0.072 | -0.3673 | Yes |
| 81 | BET1 | Bet1 golgi vesicular membrane trafficking protein | 17898 | -0.074 | -0.3572 | Yes |
| 82 | ATP6V1H | ATPase, H+ transporting, lysosomal 50/57kDa, V1 subunit H | 18008 | -0.077 | -0.3468 | Yes |
| 83 | ARCN1 | archain 1 | 18130 | -0.081 | -0.3364 | Yes |
| 84 | SEC22B | SEC22 homolog B, vesicle trafficking protein (gene/pseudogene) | 18134 | -0.081 | -0.3198 | Yes |
| 85 | YKT6 | YKT6 v-SNARE homolog (S. cerevisiae) | 18191 | -0.082 | -0.3057 | Yes |
| 86 | ICA1 | islet cell autoantigen 1 | 18218 | -0.083 | -0.2898 | Yes |
| 87 | SEC31A | SEC31 homolog A, COPII coat complex component | 18367 | -0.088 | -0.2793 | Yes |
| 88 | BNIP3 | BCL2/adenovirus E1B 19kDa interacting protein 3 | 18512 | -0.093 | -0.2675 | Yes |
| 89 | LMAN1 | lectin, mannose-binding, 1 | 18623 | -0.097 | -0.2530 | Yes |
| 90 | GOSR2 | golgi SNAP receptor complex member 2 | 19041 | -0.132 | -0.2470 | Yes |
| 91 | ATP7A | ATPase, Cu++ transporting, alpha polypeptide | 19148 | -0.149 | -0.2217 | Yes |
| 92 | ARFGAP3 | ADP-ribosylation factor GTPase activating protein 3 | 19157 | -0.151 | -0.1909 | Yes |
| 93 | SEC24D | SEC24 homolog D, COPII coat complex component | 19384 | -0.228 | -0.1553 | Yes |
| 94 | DST | dystonin | 19494 | -0.380 | -0.0823 | Yes |
| 95 | GALC | galactosylceramidase | 19500 | -0.405 | 0.0012 | Yes |
Table: GSEA details [plain text format]

  

Fig 2: HALLMARK\_PROTEIN\_SECRETION      
 Blue-Pink O' Gram in the Space of the Analyzed GeneSet

  

Fig 3: HALLMARK\_PROTEIN\_SECRETION: Random ES distribution      
 Gene set null distribution of ES for **HALLMARK\_PROTEIN\_SECRETION**
